# Uveitis-induced Refractory Ocular Hypotony Managed with High-dose Latanoprost

**DOI:** 10.18502/jovr.v15i3.7459

**Published:** 2020-07-29

**Authors:** Fariba Ghassemi, Mohammad Reza Niyousha, Narges Hassanpoor, Hassan Khojasteh

**Affiliations:** ^1^Retina & Vitreous Service, Farabi Eye Hospital, Tehran University of Medical Sciences, Tehran, Iran; ^2^Retina & Vitreous Service, Nikookari Eye Hospital, Tabriz University of Medical Sciences, Tabriz, Iran; ^3^Ophthalmic Research Center, Shahid Beheshti University of Medical Sciences, Tehran, Iran; ^4^Department of Ophthalmology, Tabriz University of Medical Sciences, Tabriz, Iran

**Keywords:** Behcet, Hypotony, Inflammation, Latanoprost, Uveitis

## Abstract

**Purpose:**

To report a case of refractory ocular hypotony due to chronic Behcet's disease with good response to high-dose topical latanoprost.

**Case Report:**

We present a 26-year-old man with a known history of Behcet's disease who developed decreasing vision and severe ocular hypotony that was refractory to multiple treatment modalities including subtenon triamcinolone acetonide, ibopamine, pars plana vitrectomy, and silicone oil injection. We decided to try high-dose topical latanoprost for the management of ocular hypotony based on recent reports. After six months, intraocular pressure (IOP) increased by 5 mm Hg, became stable at 7 mm Hg, and remained unchanged at month 24.

**Conclusion:**

High-dose topical latanoprost could lead to significant increase in IOP in uveitis-induced refractory ocular hypotony.

##  INTRODUCTION 

Ocular hypotony is one of the complications of uveitis and causes substantial visual loss. It occurs because of hyposecretion of the ciliary body owing to inflammation and increased uveoscleral outflow. In the acute phase of the disease, it is usually reversible; and suppressing the inflammation by corticosteroids or other anti-inflammatory medications may restore the intraocular pressure (IOP). However, in chronic uveitis, long-term inflammation can lead to structural changes such as growth of tractional membranes on the ciliary body and atrophic changes resulting in long-lasting or irreversible hypotony. Chronic ocular hypotony is associated with sight-threatening complications including hypotony maculopathy, choroidal folds, and optic nerve edema.^[1,2,3]^ Ocular hypotony is defined as IOP < 8 mm Hg, and most complications arise when IOP is < 4 mm Hg.^[4]^

##  CASE REPORT

In this report, we present a 26-year-old male with a past medical history of Behcet's disease who developed progressive vision loss and severe hypotonia. He had received 15 mg of methotrexate weekly and 7.5 mg of prednisolone daily as well as multiple injections of subtenon triamcinolone acetonide (TA; 40 mg). He had also undergone phacoemulsification and posterior chamber intraocular lens placement for cataract in his both eyes. Pars plana vitrectomy with silicone oil injection was performed in his right eye for hypotony. Visual acuity was 20/400 in his right eye and “hand motion” in his left eye. Ocular hypotony persisted despite all these treatments in the absence of active inflammation. Corneal folds and band keratopathy were noted after few weeks. Fundus was poorly visible but it was remarkable for cystic changes in the macular region. B-scan showed a significant serous choroidal detachment due to severe hypotony in both eyes. To increase the IOP, multiple injections of 40 mg of subtenon and 4 mg of intravitreal TA were administered; however, no improvement was observed in vision, IOP status, and serous choroidal detachment. Visual acuity deteriorated because of persistent hypotony maculopathy. Ibopamine (a dopamine agonist) eye drops were used for three months with an increase in IOP of 2 mm Hg in both the eyes, but no change in vision was detected.

We discussed the details of our experimental treatment based on published studies with the patient and proceeded with the treatment after obtaining a written consent. Subsequently, high-dose latanoprost eye drops (XALATAN, 0.005%, Pfizer) were administered every 6 hours in both eyes.

**Figure 1:**
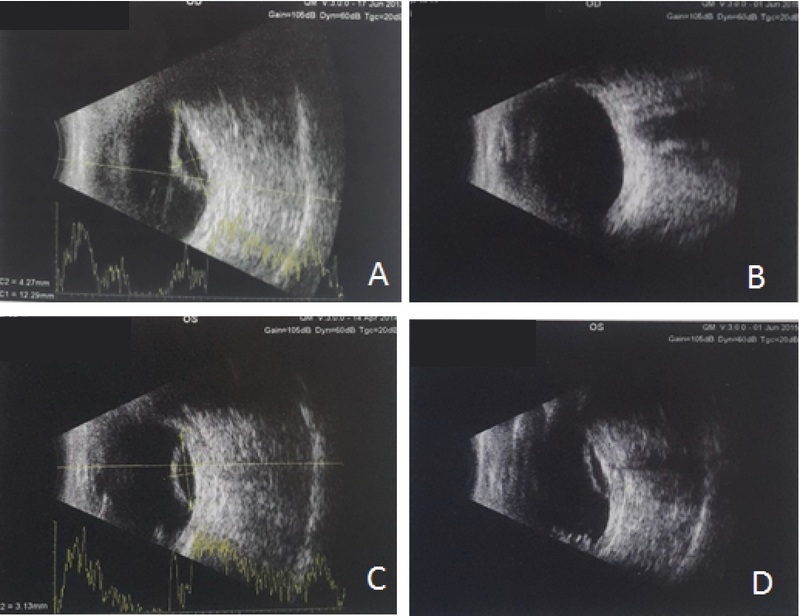
(A) Right eye B-scan before latanoprost treatment. (B) Serous choroidal detachment resolution after treatment with high-dose latanoprost in the right eye. (C) Left eye B-scan before latanoprost treatment. (D) Serous choroidal detachment improvement after treatment in the left eye.

One month later, IOP increased to 4 mm Hg, and at two months, to 7 mm Hg. After two months of latanoprost treatment, we performed a drug rechallenge test by discontinuing latanoprost for four weeks and then resuming the drug to prove its effect on IOP. After 6 months, IOP was stable at 7 mm Hg and remained unchanged even after 24 months. B-scan showed significant improvement in hypotony maculopathy and fluid resolved subretinally (Figures 1B and D). The patient's vision improved to 20/200 in his right eye and “finger counting” at 1.5 m in his left eye.

##  DISCUSSION

Chronic ciliary body inflammation leads to traction on the ciliary body, atrophic changes of this tissue, and subsequent ocular hypotony. Serous choroidal detachment can occur because of chronic hypotony. If ciliary body traction or detachment is visible on ultrasound biomicroscopy, vitrectomy and membranectomy with or without silicone oil injection may be used to improve the IOP. For our patient, although we performed a vitrectomy with silicone oil injection in the right eye, IOP did not change. In such cases, the rise in IOP may be temporary and mandate reinjection of silicone oil in approximately 50% of patients.^[1,2,3]^ Ibopamine is a dopamine agonist that can increase aqueous humor secretion and IOP. Administration of ibopamine eye drops increases the IOP by approximately 2 mm Hg.^[5]^ In our case, ibopamine improved IOP to a barely measurable 2 mm Hg. Subtenon and intravitreal injections of TA or systemic corticosteroids may increase IOP in chronic hypotony.^[6,7]^ However, these treatments did not improve results in our patient.

Prostaglandins (PGs) play an important role in aqueous humor dynamics. Latanoprost is a selective PGF${}_{2}$ receptor agonist that can reduce IOP by increasing aqueous outflow mostly by increasing the uveoscleral outflow. It is a potent antiglaucoma treatment when applied once daily.^[8]^ However, when administered topically at high concentrations or intracamerally, PGs E and F can cause miosis and raise the IOP. At the same time, they increase the protein content and white blood cells in the aqueous humor.^[9]^

In chronic uveitis, ciliary body atrophy and consequent aqueous hyposecretion may be difficult to overcome; and decreasing aqueous outflow may be a better way to restore the IOP. High-dose latanoprost may cause some grades of inflammation in trabecular meshwork and reduce the aqueous outflow. In cases with no tractional membranes on the ciliary body, it may be more practical to increase the uveoscleral outflow. This could be a mechanism by which high-dose latanoprost can raise the IOP in similar cases as our patient. Another less possible mechanism could be improvement of the serous choroidal detachment due to an increase in uveoscleral outflow.

In a report of three cases with uveitic glaucoma, latanoprost had a paradoxical impact on IOP.^[10]^ Latanoprost significantly increased IOP, and a latanoprost rechallenge test—performed by discontinuing and then continuing the drug—proved that latanoprost could increase the IOP. All patients had uveitis; thus, it can be postulated that in the presence of a severely damaged blood-ocular barrier, latanoprost can have a paradoxical impact on IOP. In that report, latanoprost was administered at a therapeutic once daily dose, and it may be assumed that higher doses, as in our case, may have a more prominent impact on IOP. Higher doses of latanoprost may result in more PG release from the impaired blood-ocular barrier and trabecular inflammation, which can overcome the increase in uveoscleral aqueous outflow and result in raised IOP.^[10]^

Latanoprost has reportedly been used in uveitic patients without the risk of central macular edema or recurrence of anterior uveitis.^[11]^ In patients with anterior and intermediate uveitis, low-dose (once daily) latanoprost did not show higher rates of inflammation recurrence when compared to a fixed combination of dorzolamide and timolol.^[12]^ However, similar findings have not been seen in patients with severe posterior uveitis due to Behcet's disease. Therefore, despite it appearing paradoxical to use latanoprost as an IOP-recovering agent in a uveitic patient, its effect might be dependent on the type, severity, and chronicity of uveitis.

It can be concluded that, in some cases, high-dose latanoprost can be administered as an adjuvant treatment for refractory hypotony due to chronic inflammation. Prospective clinical trials to further investigate this can be beneficial.

##  Conflicts of Interest

There are no conflicts of interest
